# The influence of language deprivation in early childhood on L2 processing: An ERP comparison of deaf native signers and deaf signers with a delayed language acquisition

**DOI:** 10.1186/1471-2202-13-44

**Published:** 2012-05-03

**Authors:** Nils Skotara, Uta Salden, Monique Kügow, Barbara Hänel-Faulhaber, Brigitte Röder

**Affiliations:** 1 Biologische Psychologie & Neuropsychologie, Universität Hamburg, Von-Melle-Park 11, Hamburg, 20146, Germany; 2 Erziehungswissenschaften, Sektion II: Wahrnehmung & Kommunikation, Universität Hamburg, Sedanstr. 19, Hamburg, 20146, Germany

## Abstract

**Background:**

To examine which language function depends on early experience, the present study compared deaf native signers, deaf non-native signers and hearing German native speakers while processing German sentences. The participants watched simple written sentences while event-related potentials (ERPs) were recorded. At the end of each sentence they were asked to judge whether the sentence was correct or not. Two types of violations were introduced in the middle of the sentence: a semantically implausible noun or a violation of subject-verb number agreement.

**Results:**

The results showed a similar ERP pattern after semantic violations (an N400 followed by a positivity) in all three groups. After syntactic violations, native German speakers and native signers of German sign language (DGS) with German as second language (L2) showed a left anterior negativity (LAN) followed by a P600, whereas no LAN but a negativity over the right hemisphere instead was found in deaf participants with a delayed onset of first language (L1) acquisition. The P600 of this group had a smaller amplitude and a different scalp distribution as compared to German native speakers.

**Conclusions:**

The results of the present study suggest that language deprivation in early childhood alters the cerebral organization of syntactic language processing mechanisms for L2. Semantic language processing instead was unaffected.

## Background

The majority of deaf children have hearing parents, whose primary language is a spoken language. Children born severely or profoundly deaf do not have access to a spoken language and, thus, do not acquire a language from birth. In Germany, these children usually acquire DGS from school mates after entering primary school or even later [1,2]. Deaf children who lack input from a language model in the family usually generate a gestural communication system called ‘homesign’. Such a system, however, is not a fully realized natural language and hence does not support language development [3]. By contrast, language development in deaf children of deaf parents whose primary language is a sign language develops similarly to hearing children of hearing parents [4-6]. From a linguistic point of view, sign languages are complete, natural, and fully realized languages with a phonology, morphology, syntax, and semantics [7,8]. Furthermore, studies on L1 processing have shown that neural correlates of oral language processing in native speakers and sign language processing in deaf signers are largely overlapping [9-11].

In our ERP study, we investigated the effects of a delayed L1 acquisition in a violation paradigm. Semantic violations are known to be associated with a negative ERP, the so called ‘N400 effect’ that has mostly been observed over centro-parietal electrode sites, particularly for written languages [12]. The N400 is considered to reflect lexical semantic integration processes [13,14]. Additionally, semantic violations within the arguments of the verb sometimes elicit a positive ERP as well, which has been related to the recruitment of additional resources for processing complex stimuli [13,15,16]. By contrast, syntactic processing has been associated with the left anterior negativity (LAN) and the P600 (or syntactic positive shift, SPS, [17]). The LAN occurred for example after congruency violations with a similar latency as the N400 [18-21]. The LAN seems to reflect either relatively automatic syntactic processes [22,23], and/or working memory load due to complex processing operations [24]. The P600 has been observed after various syntactic anomalies, such as morphosyntactic violations, and within garden-path sentences [21,25]. It is considered to reflect the processing costs of a re-analysing process after an anomaly detection [for example [26]].

L2 learners who are exposed to the L2 after puberty have shown lower performance and a higher variability in L2 processing compared to native speakers of the same language. A negative correlation between the age of onset of acquisition of an L2 and the achieved grammatical competence has been observed in many studies [for an overview see for example: [27,28]]. Importantly, lexical-semantic aspects of a language seem to be less affected by a late language acquisition than syntactical and phonological aspects [29].

Many studies found that the semantic effect (N400) is robust to effects of age of acquisition (AoA) [23,30], whereby the syntactic LAN effect was affected to a larger degree. For example, Weber-Fox et al. [30] found that even L2 learners with an AoA of four years did not show a native-like LAN. Chen et al. [31] investigated Chinese L2 learners of English: Despite accurate grammatical judgements for subject-verb agreement violations, they did not show a LAN but a negativity between 500–700 ms. These ERP differences between L1 and L2 learners have been suggested to reflect a reduced automatic language processing in L2 learners. In contrast to native speakers, L2 learners seem to explicitly recapitulate the words and phrases of the L2, resulting in an additional drain on working memory load [32]. These differences in language processing between L1 and L2 speakers have been explained by the hypothesis of a sensitive (SP) or critical period (CP) for language acquisition in the development of the nervous system, during which learning capabilities are enhanced. According to Knudsen’s definition [33], a CP is characterized by an abrupt loss of learning capabilities after its expiration, whereas a SP only implies a considerable decline of learning capabilities after its expiration [34]. However, apart from AoA, differences in L2 processing are influenced by the similarity between the L1 and L2 and/or the proficiency level of the participants [23,35-37]. Automatic parsing processes, for example, as reflected in the LAN, have been shown in highly proficient L2 learners in several ERP studies [23,35,38].

By comparing the L2 processing of (1) deaf people who had acquired German Sign Language (DGS) as their L1 from their deaf parents (henceforth: ESL for Early Sign Language learners), and (2) deaf people who had not been exposed to sign language during the first years of life because they had hearing parents (henceforth: LSL for Late Sign Language learners) it is possible to study the effects of a delayed L1 acquisition compared to a timely L1 acquisition in sign language users when tested in their L2 German. German processing was assessed by analysing a group of (3) hearing German native speakers (henceforth: EGL for Early German Language learners). Both groups of deaf people, ESL and LSL, started to learn German at the time of primary school enrolment.

In this regard, Mayberry and Lock [39] compared the English competence of deaf native speakers of a sign language and hearing native speakers of a spoken language with deaf people with a delayed L1 acquisition (sign language). All three groups were tested in their L2 English. Native speakers of a sign language and native speakers of a spoken language performed similarly on a high level. Interestingly, deaf people with a delayed exposure to sign language performed significantly worse than both groups of native speakers. Chamberlaine and Mayberry [40] found further evidence for the notion that strong L1 skills in a sign language can scaffold strong skills in the written representation of a spoken L2. These authors [39,40] suggest that children need the benefits of a natural language irrespectively of its modality for any successful language acquisition. By learning an L1 from birth, basic abstract principles of form and structure are acquired that are independent of the sensory motor modality through which a language is expressed. These principles create the lifelong ability to learn a language.

The effects of a delayed L1 acquisition on the neural processing mechanisms in a written L2 in sign language users have not been investigated yet. Using ERPs, we were able to assess the effects of a delayed L1-acquisition separately on semantic and syntactic aspects of language processing. Semantic ERPs are relatively robust to effects of AoA [23,30], in contrast to the LAN that often differentiates between native speakers and L2 learners: L2 learners who are not highly proficient usually do not show a LAN [30,31].

### Predictions

In accord with the findings of Mayberry and Lock [39], the LSL participants were expected to reach a lower performance level than ESL in several language tests (ATBG, see materials section) and in the task of the EEG experiment.

With regards to the ERP results of the group of EGL in accordance with previous findings in native speakers [12,21,22,25], we expected an N400 with a centro-parietal topography in the semantic condition. In the syntactic condition we expected a LAN at the left anterior clusters L1 and/or L2 [see also [41]]. The LAN was expected to be followed by a posteriorly distributed positivity (P600) in the syntactic condition.

For ESL a similar N400 effect in the semantic condition has been predicted. Previous findings have suggested that L2 learners who are neither highly proficient nor very familiar with the grammatical phenomenon do not show a LAN and/or a reduced P600 [30,37,42]. However, we recently reported both a LAN and a P600 for ESL similar to hearing L2 learners [41].

As previous studies give reason to expect differences between ESL and LSL [39], we predicted the absence of a LAN effect in LSL. Since syntactic and phonological aspects of language are generally more vulnerable to AoA effects than lexical-semantic aspects, we also expected an N400 effect in the group of LSL [29].

## Results

### Language proficiency tests (ATBG)

The language proficiency test was run with all deaf participants. One-sided t-tests showed significantly higher performance in ESL than LSL in the following subtests of the ATBG: TGK (t(21.766) = 2.219; p = 0.019), PPVT (t(24.794) = 1.865; p = 0.037), and GSV (t(22.666) = 1.822; p = 0.041). The groups did not significantly differ in the subtest ADST (t(24.703) = 0.552; p = 0.293). The subgroups of LSL and ESL who had reached the criterion of at least 60% correct sentence judgements in all conditions of the EEG experiment did not differ significantly in any of the four subtests of the ATBG (all p > 0.1). The results of the ATBG are shown in Table 1.

**Table 1:** Arithmetic means and standard errors of the percentages of correct responses of the two deaf groups in the four subtests of the ATBG

**Subtest**	**ESL**	**LSL**
TGK	85.7 (3.1)	71.7 (5.4)
PPVT	89.9 (2.2)	83.5 (2.7)
ADST	79.6 (5.2)	74.9 (6.6)
GSV	85.8 (3.2)	74.7 (5.2)

TGK: sign language comprehension, PPVT: vocabulary,

ADST: general German language, TGK: grammatical competence.

ESL: deaf early sign language learners, LSL: deaf late sign language learners.

### Behavioural data

As mentioned in the methods section, 8 out of 12 participants of the ESL group and 8 out of 15 participants of the LSL group performed at a level of at least 60% correct in all three conditions (correct, semantically incorrect, syntactically incorrect) in the EEG experiment. Here we report results of those participants who met the 60% criterion (high performing participants), since ERP violation effects are not expected for participants performing at chance level. The behavioural data is shown in Table 2.

**Table 2:** Arithmetic means and standard errors of the percentages of correct responses of the experimental groups in the three conditions of the EEG-Experiment

**Condition**	**EGL**	**ESL**	**LSL**	**ESL (n.a.)**	**LSL (n.a.)**
correct	94.7 (0.9)	92.0 (1.2)	82.5 (2.6)	92.0 (1.6)	82.1 (3.5)
semantics	97.6 (0.8)	94.2 (1.2)	94.5 (1.8)	93.1 (3.4)	84.1 (5.9)
syntax	95.5 (1.9)	77.3 (4.2)	80.5 (4.5)	27.8 (11.6)	34.3 (10.6)

EGL: hearing early German language learners, ESL: deaf early sign language learners, LSL: deaf late sign language learners, n.a.: not analyzed.

The ANOVA for the three groups revealed main effects of Group (F(2, 25) = 11.682; p < 0.001), and Condition (F(1.1, 26.3) = 20.297; ϵ = 0.525; p < 0.001), as well as an interaction between Group and Condition (F(2.1, 26.3) = 7.576; ϵ = 0.525; p < 0.001). The EGL differed significantly from LSL (t(8.096) = 3.632; p = 0.020) as well as from ESL (t(9.967) = 4.514; p = 0.003). The groups LSL and ESL did not differ (t(11.550) = 0.643; p = 0.533).

In the correct condition, LSL performed significantly worse than EGL (t(8.760) = 4.378; p = 0.006) and ESL (t(9.775) = 3.271; p = 0.026).

No group differences were observed in the semantic condition.

In the syntactic condition, EGL performed at a higher level than LSL (t(9.591) = 3.105; p = 0.035) and ESL (t(9.856) = 3.909; p = 0.009).

### EEG data

The results of the ANOVAs for each of the three groups of participants (EGL, ESL, LSL) are presented in the following sections. They are followed by between-group comparisons. Results for the semantic condition are always reported first, followed by the results for the syntactic condition. ERP effects of both the semantic and the syntactic condition were analyzed for the time epochs 300–500 ms and 600–800 ms. For the syntactic condition, the time interval of 300–500 ms was further divided into three sub-epochs of 66 ms each. The ANOVA model comprised the within-group factors Condition (CO), Hemisphere (HE), and Cluster (CL). For the between-groups ANOVAs the factor Group (GR) was added.

### Results hearing early German language learners (EGL)

#### Semantic condition

**Figure 1:**
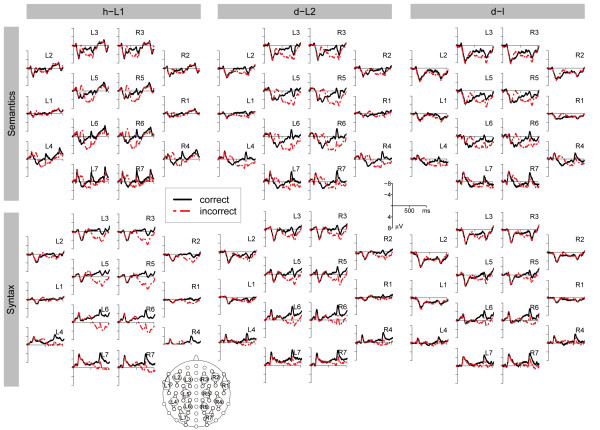
**Overview of the ERP results for all clusters.** Averaged ERPs of the semantic (first row) and syntactic (second row) condition for EGL (first column), ESL (second column), and LSL (third column) on all clusters. The dotted line denotes the ERP after the incorrect condition, the solid line the correct condition.

**Figure 2:**
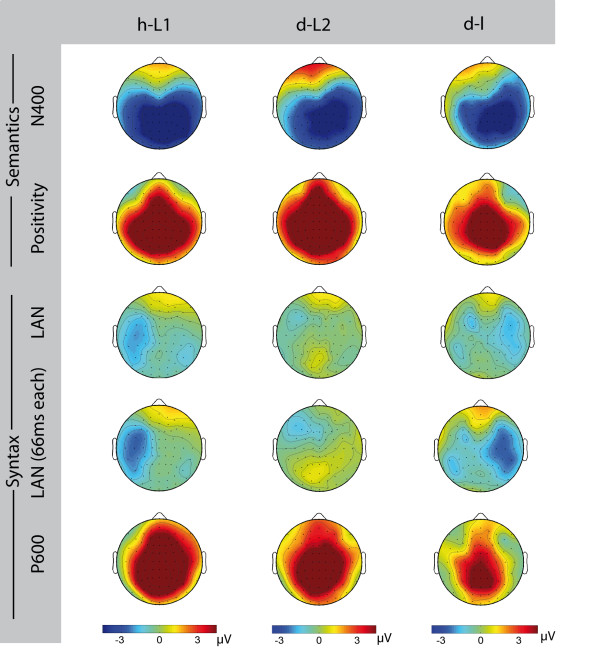
**Overview of the topographic distributions of the ERPs.** Topographies of the N400 (first row), semantic positivity (second row), LAN for 300–500 ms (third row), LAN for 66 ms each (fourth row), and P600 (fifth row) for EGL (first column), ESL (second column), and LSL (third column). Blue denotes negative differences of incorrect minus correct words and red denotes positive differences in μV. The annotation ‘66 ms each’ denotes 366-433 ms for EGL, 433–500 ms for LSL, and 300–366 ms for ESL.

In the time window of 300–500 ms, the ANOVA revealed a significant main effect of CO (F(1, 11) = 46.717; p < 0.001) and an interaction of CO and CL (F(2.3, 24.9) = 17.399; ϵ = 0.377; p < 0.001). The ERP difference between the incorrect and correct condition was negative for all clusters except L2 and L3 (p < 0.05) (see Figures 1 and 2, and Table 3).

**Table 3:** ANOVAs for the semantic condition

**Semantics**		Time epoch			
groups	effects	300–500 ms		600–800 ms	
		F	p	F	p
EGL	CO	**46.717**	**≤ 0.001**	**22.307**	**≤ 0.001**
	CO,HE	2.183	0.168	0.096	0.763
	CO,CL	**17.399**	**≤ 0.001**	**16.294**	**≤ 0.001**
	CO,HE,CL	0.545	0.612	0.373	0.660
		F	p	F	p
LSL	CO	**32.549**	**≤ 0.001**	**28.762**	**0.001**
	CO,HE	5.572	0.050	0.395	0.549
	CO,CL	**11.111**	**0.004**	**12.062**	**0.002**
	CO,HE,CL	5.239	0.020	2.257	0.166
		F	p	F	p
ESL	CO	4.943	0.062	11.999	0.010
	CO,HE	9.011	0.020	0.033	0.862
	CO,CL	**6.883**	**0.007**	5.918	0.016
	CO,HE,CL	5.416	0.026	0.296	0.741

CO: Condition, HE: Hemisphere, CL: Cluster; p ≤ 0.05; p ≤ **0.01**; p ≤ **0.001**; EGL: hearing early German language learners, ESL: deaf early sign language learners, LSL: deaf late sign language learners.

For 600–800 ms, the incorrect condition was significantly more positive than the correct condition (F(1, 11) = 22.307; p = 0.001). The interaction of CO and CL (F(1.8, 19.9) = 16.294; ϵ = 0.302; p < 0.001) was significant as well. The ERP difference for incorrect and correct words was positive for all clusters except L1 and R2 (p < 0.05) (see Figures 1 and 2, and Table 3).

#### Syntactic condition

**Figure 3:**
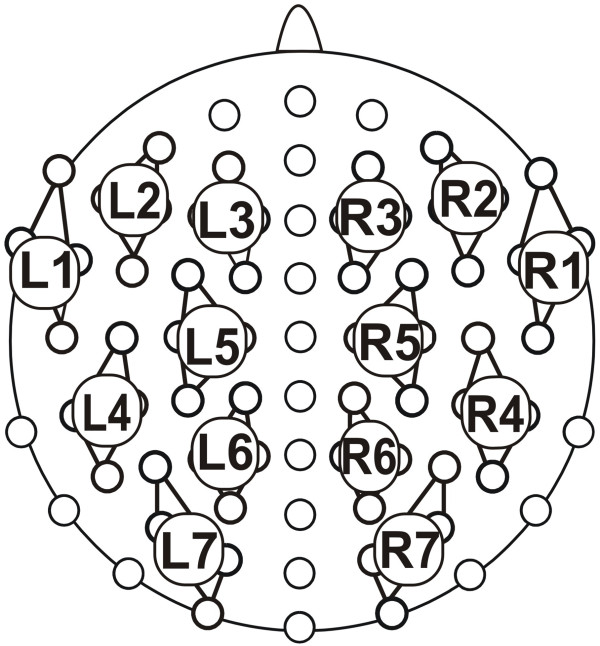
**Overview of the ERP results for selected clusters.** Averaged ERPs in the semantic (first row) and syntactic condition (second row) for EGL (first column), ESL (second column), and LSL (third column) on clusters L5 (semantics) and L1 (syntax). The dotted line denotes the ERP after the incorrect condition, the solid line the correct condition.

An interaction of CO and HE (F(1, 11) = 11.162; p = 0.007) was observed for the time window of 300–500 ms, indicating a stronger violation effect over the left than over the right hemisphere. The difference between the incorrect and the correct condition was significant for clusters L1, L2, L4, L5, and R4 (p < 0.05) (see Figures 2 and 3). The results of the three sub time epochs did not reveal a significant violation effect between 300–366 ms. By contrast, the following significant effects were obtained for the second and third time epoch: 366–433 ms: interactions of CO and HE (F(1, 11) = 23.788; p < 0.001), and CO, HE, and CL (F(2.1, 23.6) = 4.899; ϵ = 0.358; p = 0.015); 433–500 ms: a main effect of CO (F(1, 11) = 6.605; p = 0.026), and an interaction of CO and CL (F(2.2, 23.8) = 5.191; ϵ = 0.360; p = 0.012) (see Figures 2 and 3).

**Figure 4:**
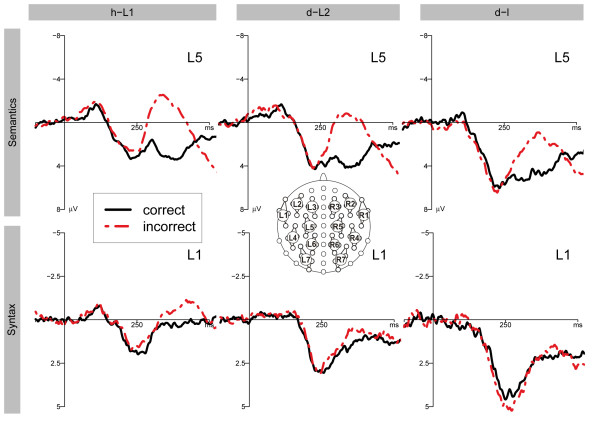
**Electrode montage and clustering.** Four adjacent electrodes each were averaged into the 14 marked clusters, seven over the left (clusters L1–L7) and seven over the right (clusters R1–R7) hemisphere.

The ANOVA for 600–800 ms revealed a main effect of CO (F(1, 11) = 38.299; p < 0.001), interactions of CO and HE (F(1, 11) = 8.541; p = 0.014), and of CO and CL (F(2.3, 24.8) = 39.924; ϵ = 0.376; p < 0.001). The latter indicated a stronger ERP effect over the right hemisphere. The difference between the incorrect and correct condition was significant for clusters L2-7 and for R1-7 (p < 0.05) (see Figures 1 and 2, and TableS 4 and 5).

**Table 4:** ANOVAs for the syntactic condition

Syntax		Time epoch			
groups	effects	300–500 ms		600–800 ms	
		F	p	F	p
EGL	CO	4.414	0.059	**38.299**	**≤ 0.001**
	CO,HE	**11.162**	**0.007**	8.541	0.014
	CO,CL	2.016	0.156	**32.924**	**≤ 0.001**
	CO,HE,CL	2.473	0.106	3.939	0.043
		F	p	F	p
LSL	CO	3.102	0.122	7.635	0.028
	CO,HE	1.608	0.245	0.036	0.855
	CO,CL	1.479	0.258	**11.768**	**≤ 0.001**
	CO,HE,CL	0.967	0.417	0.281	0.851
		F	p	F	p
ESL	CO	0.052	0.826	8.865	0.021
	CO,HE	1.076	0.334	0.241	0.638
	CO,CL	0.185	0.838	4.889	0.031
	CO,HE,CL	1.682	0.229	0.681	0.539

CO: Condition, HE: Hemisphere, CL: Cluster; p ≤ 0.05; p ≤ **0.01**; p ≤ **0.001**;EGL: Hearing Early German Language Learners, ESL: Deaf Early Sign Language Learners, LSL: Deaf Late Sign Language Learners.

**Table 5:** ANOVAs for three time epochs of the LAN in the syntactic condition

**LAN**		Time epoch					
groups	effects	300–366 ms		366–433 ms		433–500 ms	
		F	p	F	p	F	p
EGL	CO	1.642	0.226	2.303	0.157	6.605	0.026
	CO,HE	3.056	0.108	**23.788**	**≤ 0.001**	1.755	0.212
	CO,CL	0.484	0.587	1.130	0.343	5.191	0.012
	CO,HE,CL	1.434	0.260	4.899	0.015	1.133	0.344
		F	p	F	p	F	p
LSL	CO	0.041	0.845	3.619	0.099	3.085	0.122
	CO,HE	0.213	0.658	0.175	0.688	4.408	0.074
	CO,CL	0.649	0.517	1.572	0.239	1.776	0.201
	CO,HE,CL	1,314	0.298	0,558	0.617	1.441	0.260
		F	p	F	p	F	p
ESL	CO	0.026	0.877	0.330	0.584	<0.001	0.999
	CO,HE	6.487	0.038	0.006	0.939	0.146	0.714
	CO,CL	1.403	0.279	0.333	0.716	0.472	0.637
	CO,HE,CL	3.551	0.065	1.642	0.230	0.469	0.575

CO: Condition, HE: Hemisphere, CL: Cluster; p ≤ 0.05; p ≤ **0.01**; p ≤ **0.001**;EGL: Hearing Early German Language Learners, ESL: Deaf Early Sign Language Learners, LSL: Deaf Late Sign Language Learners.

### Summary: Hearing early German language learners (EGL)

In sum, in the semantic condition, EGL showed an N400 with a typical symmetric centro-parietal distribution and a subsequent symmetrically distributed positivity with a peak at posterior sites. The syntactic condition elicited a LAN with a left-temporal distribution that was followed by a right lateralized medially distributed P600.

### Results early sign language learners (ESL)

#### Semantic condition

In the time window of 300–500 ms, the ANOVA revealed significant interactions of CO and CL (F(2.1, 14.7) = 6.883; ϵ = 0.351; p = 0.007), and of CO and HE (F(1, 7) = 9.011; p = 0.020), and of CO, HE, and CL (F(1.7, 11.7) = 5.416; ϵ = 0.278; p = 0.026), indicating a smaller ERP difference over the left than over the right hemisphere. Moreover, the interaction of CO, HE, and CL (F(6, 54) = 7.486; ϵ = 0.506; p = 0.001) was significant (negative differences at L6, L7, and R1, R4, R5, R6, R7; p < 0.05) (see Figures 1 and 2, and Table 3).

For the time epoch 600–800 ms, the incorrect condition was significantly more positive than the correct condition (F(1, 7) = 11.999; p = 0.010). The interaction of CO and CL (F(1.9, 13.3) = 5.918; ϵ = 0.316; p = 0.016) was significant, as well. The t-tests revealed positive ERP differences between incorrect and correct conditions for clusters L3-7, and R3-7 (p < 0.05) (see Figures 1 and 2, and Table 3).

#### Syntactic condition

There was no significant effect for time epoch 300–500 ms. However, a significant interaction of CO, and HE (F(1, 7) = 6.487; p = 0.038) was found for time epoch 300–366 ms, in which the violation effect was significant for cluster L1 and L2 (p < 0.05) (see Figure 3 and Table 5). No further significant effects were observed in the time epochs 366–433 ms and 433–500 ms.

Between 600–800 ms, a main effect of CO (F(1, 7) = 8.865; p = 0.021) and an interaction of CO and CL (F(1.7, 12.2) = 4.889; ϵ = 0.291; p = 0.031) were observed. The positive difference between the incorrect condition and the correct condition was significant for clusters L3-7 and R3-7 (p < 0.05) (see Figures 1 and 2, and TableS 4 and 5).

### Summary early sign language learners (ESL)

Taken together, ESL showed an N400 to semantic violations, which was more pronounced over the right than the left hemisphere. The N400 was followed by a symmetrically distributed positive ERP effect.

A LAN was observed after syntactic violations, which was significant for a sub-epoch of the analysed LAN time window. Additionally, a symmetrically distributed P600 was observed in the syntactic condition.

### Results late sign language learners (LSL)

#### Semantic condition

A main effect of CO (F(1, 7) = 32.594; p < 0.001), and interactions of CO and CL (F(1.5, 10.3) = 11.111; ϵ = 0.245; p = 0.004) and of CO, HE, and CL (F(2.0, 13.9) = 5.239; ϵ = 0.330; p = 0.020) were significant in the time window of 300–500 ms after the onset of the critical word (see Figures 1 and 2, and Table 3). The difference between incorrect and correct conditions was negative. Clusters L4-7 and R1-7 showed significantly more negative amplitudes after an incorrect than a correct critical word (p < 0.05).

For 600–800 ms, a main effect of CO (F(1, 7) = 28.762; p = 0.001) and an interaction of CO and CL (F(1.6, 11.6) = 12.062; ϵ = 0.275; p = 0.002) reached significance. The difference between incorrect and correct conditions was positive (see Figures 1 and 2, and Table 3). A significant positive difference was observed for clusters L3-7 and R3-7 (p < 0.05).

#### Syntactic condition

Neither the ANOVA for the time window 300–500 ms nor the ANOVAs for the three 66 ms time epochs revealed any significant effect. A marginally significant CO and HE interaction was found for the last of the three intervals (433–500 ms; F(1, 7) = 4.408; p = 0.074). This interaction was due to a more negative violation effect over the right hemisphere (see Figure 2). The t-tests for single clusters revealed a significant negative difference between incorrect and correct conditions for Clusters R2, R4, and R5 (p < 0.05; for the time window 300–500 ms; see Figures 1 and 2, and TableS 4 and 5).

In the interval of 600–800 ms, the main effect of CO (F(1, 7) = 7.635; p = 0.028), and the interaction of CO and CL (F(2.5, 17.2) = 11.768; ϵ = 0.410; p < 0.001) were significant. Clusters L3, L5, L6, L7, and R5, R6, and R7 showed a more positive ERP in the incorrect condition than in the correct condition (see Figures 1 and 2, and TableS 4 and 5).

### Summary late sign language learners (LSL)

An N400 as well as a semantic positivity were observed in LSL. Syntactic violations did not elicit a significant LAN effect. A P600 effect was observed following syntactic violations within 600–800 ms.

### Group comparisons

#### Semantic condition

In the ANOVAs for the time epochs 300–500 ms and 600–800 ms including all three possible pairs of groups, none of the effects involving GR and CO was significant (see Figure 2 and Table 6).

**Table 6:** Group comparisons for the semantic condition

**Group comparisons Semantics**		Time epoch			
groups	effects	300–500 ms		600–800 ms	
		F	p	F	p
EGL/LSL	GR,CO	0.954	0.342	0.928	0.348
	GR,CO,HE	1.498	0.237	0.575	0.458
	GR,CO,CL	0.511	0.614	0.647	0.519
	GR,CO,HE,CL	2.209	0.114	1.793	0.185
		F	p	F	p
EGL/ESL	GR,CO	0.360	0.556	0.357	0.558
	GR,CO,HE	1.687	0.210	0.107	0.747
	GR,CO,CL	0.086	0.936	0.222	0.792
	GR,CO,HE,CL	2.485	0.088	0.519	0.619
		F	p	F	p
LSL/ESL	GR,CO	0.000	0.993	1.760	0.206
	GR,CO,HE	0.004	0.950	0.066	0.802
	GR,CO,CL	0.169	0.857	0.257	0.768
	GR,CO,HE,CL	0.172	0.848	0.407	0.654

GR: Group, CO: Condition, HE: Hemisphere, CL: Cluster; p ≤ 0.05; p ≤ **0.01**; p ≤ **0.001**;EGL: Hearing Early German Language Learners, ESL: Deaf Early Sign Language Learners, LSL: Deaf Late Sign Language Learners.

#### Syntactic condition

For the time epoch of 300–500 ms, the interaction of GR, CO, and HE (F(2, 25) = 5.368; p = 0.011 was significant in the ANOVA including all three groups.

The comparison of EGL and LSL resulted in a significant GR, CO, and HE interaction (F(1, 18) = 8.986; p = 0.008) that was due to a negative difference over the left hemisphere for EGL and a marginally significant difference (see above results for group LSL) over the right hemisphere for LSL (see Figure 2, and Table 7).

**Table 7:** Group comparisons for the syntactic condition

**Group comparisons Syntax**		Time epoch			
groups	effects	300–500 ms		600–800 ms	
		F	p	F	p
EGL/LSL	GR,CO	0.006	0.940	5.906	0.026
	GR,CO,HE	**8.986**	**0.008**	3.748	0.069
	GR,CO,CL	0.849	0.450	2.718	0.066
	GR,CO,HE,CL	2.227	0.108	2.887	0.065
		F	p	F	p
EGL/ESL	GR,CO	0.597	0.450	0.545	0.470
	GR,CO,HE	2.775	0.113	3.222	0.089
	GR,CO,CL	0.817	0.460	1.107	0.342
	GR,CO,HE,CL	0.360	0.701	1.044	0.364
		F	p	F	p
LSL/ESL	GR,CO	0.397	0.539	1.364	0.262
	GR,CO,HE	2.600	0.129	0.175	0.682
	GR,CO,CL	0.277	0.780	0.358	0.750
	GR,CO,HE,CL	1.624	0.208	0.776	0.515

GR: Group, CO: Condition, HE: Hemisphere, CL: Cluster; p ≤ 0.05; p ≤ **0.01**; p ≤ **0.001**;EGL: hearing early German language learners, ESL: Deaf Early Sign Language Learners, LSL: Deaf Late Sign Language Learners.

For the Group comparisons we selected the time epochs within the LAN time window for which we observed significant (or marginally significant in case of LSL) violation effects (see above: single group analyses). Therefore, we used time epoch 366–433 ms for the EGL group, time epoch 300–366 ms for the ESL group, and time epoch 433–500 ms for the LSL group. The ANOVAs revealed significant differences between all three pair of groups (see Figure 2 and Table 8):

**Table 8:** Group comparisons for three time epochs of the LAN in the syntactic condition

**Group comparisons LAN**		**Time epoch**							
groups	effects	300–366 ms		367–432 ms		433–500 ms		66 ms each	
		F	p	F	p	F	p	F	p
EGL/LSL	GR,CO	0.282	0.602	0.168	0.687	0.023	0.881	0.120	0.732
	GR,CO,HE	1.420	0.249	7.835	0.012	5.235	0.034	**21.322**	
	GR,CO,CL	0.273	0.732	1.023	0.376	1.035	0.375	0.273	0.808
	GR,CO,HE,CL	1.454	0.244	2.281	0.100	2.111	0.119	**5.296**	**0.003**
		F	p	F	p	F	p	F	p
EGL/ESL	GR,CO	0.330	0.573	0.107	0.748	1.215	0.285	0.740	0.401
	GR,CO,HE	0.743	0.400	9.504	0.006	0.581	0.456	5.671	0.028
	GR,CO,CL	0.990	0.372	0.690	0.531	0.627	0.550	1.934	0.155
	GR,CO,HE,CL	0.725	0.487	1.370	0.266	0.204	0.825	0.619	0.551
		F	p	F	p	F	p	F	p
LSL/ESL	GR,CO	0.001	0.975	0.379	0.548	0.693	0.419	1.290	0.275
	GR,CO,HE	2.125	0.167	0.162	0.693	3.601	0.079	8.493	0.011
	GR,CO,CL	0.765	0.465	0.188	0.855	0.309	0.756	2.283	0.115
	GR,CO,HE,CL	2.362	0.097	0.551	0.630	1.405	0.261	3.987	0.017

GR: Group, CO: Condition, HE: Hemisphere, CL: Cluster; p ≤ 0.05; p ≤ **0.01**; p ≤ **0.001**;EGL: Hearing Early German Language Learners, ESL: Deaf Early Sign Language Learners, LSL: Deaf Late Sign Language Learners.

EGL and LSL: GR*CO*HE: F(1, 18) = 21.322; p < 0.001; GR*CO*HE*CL: F(2.8, 51.0) = 5.296; ϵ = 0.472; p = 0.003.

EGL and ESL: GR*CO*HE: F(1, 18) = 5.671; p = 0.028.

ESL and LSL: GR*CO*HE: F(1, 14) = 8.493; p = 0.011; GR*CO*HE*CL: F(2.7, 38.5) = 3.987; ϵ = 0.458; p = 0.017.

These interactions show that LSL displayed a right lateralized topography whereas the syntactic violation effect was negative over the left hemisphere in EGL and ESL.

In the time window of 600–800 ms, the ANOVA for all three groups did not reveal any significant effect involving the factors Group and Condition. However, the direct comparison of EGL and LSL resulted in a significant GR and CO interaction (F(1, 18) = 5.906; p = 0.0258). A stronger violation effect was present in EGL compared to LSL. The ANOVAs of the other pairwise group comparisons - EGL and ESL, and ESL and LSL - did not reveal any significant effects (see Figure 2, and Table 7).

### Summary group comparisons

The group comparisons revealed no significant difference in the semantic condition.

The three time epochs of 66 ms each in which EGL and ESL showed a significant and LSL a marginally significant effect were chosen to enable a direct comparison of the topographies. This comparison confirmed a significantly different scalp topography of the syntactic violation effect for LSL compared to EGL and ESL. Furthermore, LSL revealed a significantly smaller P600 effect than EGL.

## Discussion

In order to investigate the effects of a delayed L1 acquisition on the functional organization of an L2, deaf participants who had learned both written German and DGS (German Sign Language) at the time of school enrolment (LSL) were compared to native signers who had learned written German at a comparable point in time but sign language from birth (ESL). Both groups were compared to a control group of hearing German native speakers (EGL).

Only participants who performed above chance level were included in the analyses. All three groups showed an N400 effect to semantic violations that was followed by a broadly distributed positivity. These findings are in line with results of Neville et al. [43], who reported that the N400 was highly similar though slightly prolonged in deaf native signers of ASL compared to hearing native speakers in English. Our study adds to this report the finding that even deaf people with a delayed L1 acquisition displayed an N400 effect that was indistinguishable from the N400 of both German native speakers and native signers of German sign language. In accord with results from studies investigating AoA effects on semantic processes of the L2 [23,30] our results suggest that the acquisition of semantic aspects of a language are not linked to a SP within the first years of life. This conclusion is supported by the observation that deaf children of hearing parents spontaneously produce semantically meaningful gestures even in an environment in which nobody is able to sign [3].

In the syntactic condition, EGL displayed a left temporally distributed negativity which has been considered as an index of early automatic processing [22]. Interestingly, we observed a similar though overall weaker negativity to syntactic violations in the ESL [see also: [41]. Thus, it might be speculated that the acquisition of a sign language, as a fully developed natural language, might have resulted in the establishment of brain systems important to process the syntax of a human language within the most sensitive developmental periods.

By contrast, we did not find a LAN like effect for the LSL. This finding is particularly interesting given the behavioural results. Both selected deaf groups did not differ in their performance neither in the syntactic condition of the EEG experiment nor in the corresponding subtests of the ATBG. These results suggest that even signers with a delayed L1 acquisition who have achieved a relatively high performance level do not show a cerebral organization of syntactic language aspects comparable to people who have grown up with a natural language.

Across all participants of both deaf groups, LSL showed lower performances than ESL. This finding together with the ERP results supplement the observations of Mayberry and Lock [39], providing evidence for a higher L2 competence in deaf native signers compared to deaf people with a late exposure to sign language. Thus, access to a natural language, be it spoken or signed, seems to be a prerequisite for the acquisition of syntactic aspects of a written L2. These findings argue against the so called interference hypothesis [44], which postulates that acquiring an L1 occupies the neural systems for language processing, thus preventing a proper L2 acquisition. Hence, a delayed L1 acquisition should be an advantage for L2 language acquisition in relation to a timely L1 acquisition. Our results are inconsistent with this prediction.

It should be noted that seven out of fifteen participants from the LSL group and four out of twelve participants of the ESL group were not able to perform the EEG task above chance level. The drop-out rates for the two deaf groups did not differ significantly (chi-square-test: p = 0.76). Even among the best performing participants both groups of deaf signers performed worse compared to hearing German native speakers. This general disadvantage of the deaf might be partially due to overall effects of late acquisition, the available impoverished German language input [see [41] for a discussion], or the educational situation of deaf people in Germany. In the generation of our participants, the ideal of articulation practice and lip reading drills dominated the classroom instead of sign language usage [1,45-47]. The impoverished opportunities to learn written German might have contributed to the overall lower grammatical competence of deaf participants.

## Conclusion

In summary, semantic aspects of an L2 seem to be attainable regardless of age of onset of L1 acquisition. By contrast, the cerebral organization of syntactic language aspects was shown to be highly vulnerable to a delayed L1 acquisition. Thus, the opportunity to learn a natural language with all of its syntactic complexity seems crucial for the acquisition of further languages later in life.

## Methods

The research was carried out in compliance with the Helsinki Declaration. The ethics committee of the German Society for Psychology (DGPS) approved the study (reference number: BRBHF 07022008).

### Participants

Three groups of volunteers participated: (1) Congenitally profoundly deaf people who had learned DGS as their L1 and written German as an L2 (ESL), (2) deaf people who had learned DGS and written German (L2) at the time of school enrolment (LSL), and (3) hearing German native speakers (EGL). All participants were right handed and had a normal or corrected to normal vision. They received a monetary compensation of 7 € per hour. Participants of all three groups gave their voluntary informed consent before taking part in the EEG experiment. Participants with less than 60% correct responses in any condition of the EEG experiment were excluded. The 60% boundary (48 out of 80 correct responses) is the minimum level that is considered above chance level according to a one sided binomial test (p < 0.05). Participants who performed at chance level did not show ERP differences for correct and incorrect sentences.

(1) Of all twelve ESL participants (5 males; mean age: 27 years, median: 26 years, range: 20–40 years), eight ESL were included in the ERP analysis (4 males; mean age: 28 years, median: 26 years, range: 21–40 years), and four ESL were excluded from the ERP analysis since they gave less than 60% correct responses in at least one condition (3 male; mean age: 24 years, median: 24.5 years, range: 20–26 years). Of the ESL, four participants (one was included) had a ‘Realschulabschluss’, which corresponds approximately to an O-level and is usually attained after 10 years at school, seven (six were included) had ‘Abitur’, which corresponds approximately to an A-level and is usually attained after 13 years at school, and one included person did not report his educational level.

(2) Of the fifteen LSL participants (8 males; mean age: 30 years, median: 28 years, range: 19–51 years), eight were included in the ERP analysis (4 males; mean age: 28 years, median: 29.5 years, range: 19–38 years), and seven LSL were not included in the ERP analysis since they gave less than 60% correct responses in at least one condition (4 males; mean age: 31 years, median: 25 years, range: 21–51 years). Six participants (four included) of LSL had ‘Abitur’, seven (two included) a ‘Realschulabschluss’, one (included) had a university degree and one of the included participants did not report his educational level.

(3) Of the twelve EGL (3 males; mean age: 31 years, median: 28 years, range: 22–53 years), all participants could be included in the analysis. One participant of EGL had a ‘Realschulabschluss’, nine EGL had ‘Abitur’, and two had a university degree.

All participants of ESL had a hearing loss of more than 85 dB except for one participant whose ERPs were not analyzed. Their deafness was due to hereditary causes. All but one ESL had deaf siblings, one had hearing siblings (included). All participants of LSL had a hearing loss of more than 85 dB except for one participant whose ERPs were analyzed. The causes of deafness were unknown in all cases. Participants of LSL had hearing siblings except for three participants who had deaf siblings. They were in the group whose ERPs were analyzed. Both groups of deaf participants, ESL and LSL, started learning written German at the time of school enrolment when they were 6 or 7 years old. An overview of sex, age, age of German acquisition, and education (in years) of the included participants is given in Table 9.

**Table 9:** Overview of the sex, age, AoA, and education (in years) of the included participants

groups	sex	mean age	AoA German	education
EGL	3 m, 9 f	31 years (range: 22–53 years)	from birth	9 A, 1 O, 2 U
ESL	4 m, 4 f	28 years (range: 21–40 years)	6 or 7 years	6 A, 1 O, 1 nr
LSL	4 m, 4 f	28 years (range: 19–38 years)	6 or 7 years	4 A, 2 O, 1 U, 1 nr

AoA: Age of acquisition, m: male, f: female, r: right, l: left, b: both, A: Abitur, O: Realschulabschluss, U: university degree, nr: not reported; EGL: hearing early German language learners, ESL: deaf early sign language learners, LSL: deaf late sign language learners.

### Material

Prior to the EEG Experiment further language tests designed for German deaf people out of a comprehensive vocational testing battery (ATBG: ‘Aachener Testverfahren zur Berufseignung von Gehörlosen’, English: ‘Aachen’s vocational testing for the deaf’) were applied to access language abilities in German and in DGS. The two groups of deaf participants were compared in four language subtests of the ATBG:

GSV: comprehension of DGS,

PPVT: comprehension of German written vocabulary,

ADST: comprehension of German written inflectional morphology,

TGK: grammatical competence in written German.

We analyzed these tests separately using them as measures of language specific abilities. We did not treat the ATBG as a composite score. The ATBG subtests allow for an investigation of aspects of DGS and German. The ATBG performance was not used to decide which participant’s EEG data could be analyzed. For this purpose, they had to correctly judge 60% of the sentences in each condition in the EEG experiment. The subtests of the ATBG are now described in more detail:

GSV ‘Gebärdensprach-Verständnis-Test’

The ‘sign language comprehension test’ was applied to investigate the receptive ability to comprehend DGS. Five signed stories were presented in ascending degrees of difficulty. Each item contained four statements related to the plot. After each item, participants were asked to choose the single correct statement via mouse click.

PPVT ‘Peabody Picture Vocabulary Test’

This subtest measures the passive written vocabulary in German. For each item, the participants saw a written word to which they were asked to find the fitting picture out of a total of four presented pictures.

ADST ‘Allgemeiner Deutscher Sprachtest’

Initially designed for hearing fourth to fifth graders, it measures the processing of inflections in German (subtest ‘general German language test’). Each item contains a pronoun, article, adjective or verb in its basic form/infinitive. The participant’s task was to compose the correctly inflected form in a sentence context.

TGK ‘Test zur Grammatischen Kompetenz‘

In the ‘grammatical competence test’, participants were asked to build as many meaningful written German sentences out of five presented words as possible, each time with a different word order.

The material for the EEG experiment consisted of written German sentences with the following structure: (1) article, noun (subject)/(2) verb (predicate)/(3) article, noun (direct object)/(4) preposition, [an optional article], noun (prepositional phrase). Prepositional phrases consisted of either two (‘im Stall’, English: ‘in (the) stall’) or three words (‘in der Oper’, English: ‘at the opera’). Each sentence was presented in three different conditions: (1) correct, (2) containing a semantic violation (the critical word was an implausible object), or (3) containing a syntactic violation (subject-verb number agreement violation at the verb as the critical word). Example sentences for each condition are listed in Table 10.

**Table 10:** Sentence examples for each experimental condition

**Condition**	**Example sentence**
Correct	Der Mann *kocht* das Essen in der Küche. Engl.: The man *cooks* the meal in the kitchen.
Syntactic verb-agreement violation	*Der Mann *kochen* das Essen in der Küche. Engl.: *The man *cook* the meal in the kitchen.
Semantic violation	*Der Mann kocht das in der Küche. Engl.: *The man cooks the picture in the kitchen.

The critical word in an incorrect sentence had a counterpart of the same word class in its companion correct sentence at the same position. In order to reduce possible confounding influences of linear distance between subject and verb, phrase structure boundaries, working memory load, or decision and motor processes [48], we used violations in the middle of the sentence. A pilot study was conducted to assess the cloze probability of the direct objects. In the pilot study, all sentences were presented up to the article (‘Der Mann kocht das …’, English: ‘The man cooks the …’) to a group of 104 university students. They were asked to complete the sentences. Only sentences with objects which had a cloze probability of at least 50% (mean: 82.39%, sd: 14.35%) were included in the final sample. Semantic violations were generated by inserting implausible direct objects of other sentences by exchanging them across sentences.

Eighty different sentences were generated comprising two sets of 40 sentences each (see Table 10). The participants saw the sentences either in version A or in version B. Both sentences of version A and version B were divided into two different sets: sentences with the numbers 1–40 and sentences with the numbers 41–80. Each sentence in each set was presented once syntactically incorrect, once semantically incorrect, and twice correctly (once with the subject in singular and once with the subject in plural). In set A1, correct sentences with a plural subject were syntactically violated and sentences with a singular subject were semantically violated, and vice versa: In A2, correct sentences with a singular subject were syntactically violated and those with a plural subject were semantically violated. The correct sentences in version A1 (A2 respectively) were the same as the correct sentences in version B1 (B2 respectively). However, the violations in B1 resembled those of A2 (B2 and A1 respectively). The assignment of sentences to participants and conditions is given in Table 11.

**Table 11:** Assignment of sentences to participants and conditions (n = 40 in each cell)

	Version A				Version B			
Set	Set 1 (A1)		Set 2 (A2)		Set 1 (B1)		Set 2 (B2)	
Correct	singular	plural	singular	plural	singular	plural	singular	plural
Violation	semantic	syntactic	syntactic	semantic	syntactic	semantic	semantic	syntactic
	singular	plural	singular	plural	singular	plural	singular	plural

Additionally, 80 filler sentences - half of which were correct and half of which had different types of semantic and syntactic violations at varying positions - were presented. Thus, each participant saw a total of 400 sentences.

### Procedure

The German proficiency test for the deaf (ATBG) was accomplished by the two groups of deaf participants in a different session. The test was presented on a computer monitor. The instructions were part of the test software. They were given in DGS and in written German.

In the EEG experiment, the sentences were presented in random order with black letters against a grey background. The vertical visual angle was 1.53°. First, a centred fixation cross appeared for 600 ms, followed by the successive presentation of the words at a rate of 600 ms, followed by a grey screen also for 600 ms after which the presentation of two smileys, one happy and one sad, informed the participants to press one of two buttons with their index fingers, indicating whether the sentence was correct or incorrect. Half of the participants responded on the left side for a correct sentence and on the right side for an incorrect sentence, the other half in the reverse order. The participants had to press one of the buttons to go on with the experiment. Five blocks with 80 sentences each were presented. A short break was given between blocks. The experiment lasted for about 60 min.

### EEG recording

The electroencephalogram was recorded with 74 scalp electrodes, which were arranged into an elastic cap (Easy Cap; FMS, Herrsching-Breitbrunn, Germany) according to the international 10/10 system. The electrode on the right earlobe was used as the recording reference. An averaged right/left earlobe reference was calculated offline. Electrode impedance was kept below 5 kΩ. The vertical electrooculogram (VEOG) was measured with two electrodes, one placed under each eye, and recorded against the reference electrode. Horizontal eye movements were monitored using electrodes F9 and F10.

Three BrainAmp DC amplifiers with 32 channels each (Brain Products GmbH, Gilching, Germany) were used. The recorded data was digitally stored with the BrainVision Recorder software (Brain Products GmbH, Gilching, Germany), in which the analog EEG signal was sampled at 5000 Hz, filtered online with a bandpass of 0.1 to 250 Hz, and downsampled to 500 Hz for storing. Offline, the signal was filtered (high cut-off at 40 Hz, 12 dB/oct). Four adjacent electrodes were pooled into clusters, resulting in seven clusters on each hemisphere (see Figure 4). ERPs were averaged in the time periods between 100 ms before and 1500 ms after the onset of the critical words. All trials followed by an erroneous response and trials containing artefacts due to ocular movement or other extensive muscle activity were excluded. Remaining segments were baseline corrected to a 100 ms period preceding the onset of the critical word for the following conditions: (1) semantically correct, (2) semantically incorrect, (3) syntactically correct, and (4) syntactically incorrect. Separate averages were calculated for each participant.

### Data analysis

Each subtest of the ATBG was analysed by means of t-tests of the relative frequencies of correct answers between ESL and LSL that were corrected with the Welch algorithm to adjust for unequal variances. Reaction times were not analysed since participants responded much later than the onset of the violation. For the analysis of the behavioural data, a repeated measurements ANOVA was applied to the selected participants. The between participant factor Group (EGL, ESL, and LSL) and the within participant factor Condition (correct, semantically incorrect, and syntactically incorrect) were predictor variables, the percentage of correct answers the dependent variable. Post-hoc t-tests were applied to compare each possible pair of Groups. The degrees of freedom from the t-tests were corrected using the Welch algorithm. Additionally, the p-values of these t-tests were Bonferroni corrected.

For EEG data of the time periods 300–500 ms and 600–800 ms the mean amplitudes were analysed by repeated measurements ANOVAs, separately for semantics and syntax. For syntax the interval 300–500 ms was further divided into three sub segments 66 ms each. Repeated measurement factors Condition (CO: correct vs. incorrect), Hemisphere (HE: left vs. right), and Cluster (CL: one to seven) and the between participant factor Group (EGL, h-L2, ESL) were included. Sums of Squares of Type II were calculated. To compensate for violations of the assumption of sphericity in multi-channel electroencephalographic data, the Greenhouse and Geisser correction was applied and the corresponding Greenhouse/Geisser Epsilons (ε) were reported for the F-tests. Statistically significant effects without the factor Condition are not reported. T-tests for the difference between the correct and the incorrect condition at each cluster were additionally applied to add information about the topographical distribution of the effect. The open source statistical programming language ‘R’ was used for statistical analyses. The data of EGL and ESL have been previously published in a different context [41].

## Competing interests

The authors declare that they have no competing interests.

## Author’s contributions

NS, MK, BHF, and BR designed the experiment. NS and MK run the ERP experiments. NS and BR analyzed the data. NS, US, MK, BHF, and BR wrote the paper. All authors read and approved the final manuscript.
